# Assessment and Intervention for Diabetes Distress in Primary Care Using Clinical and Technological Interventions: Protocol for a Single-Arm Pilot Trial

**DOI:** 10.2196/62916

**Published:** 2025-03-31

**Authors:** Marisa Kostiuk, Susan L Moore, E Seth Kramer, Joshua Felton Gilens, Ashwin Sarwal, David Saxon, John F Thomas, Tamara K Oser

**Affiliations:** 1 Department of Family Medicine School of Medicine University of Colorado Aurora, CO United States; 2 Colorado School of Public Health Department of Community & Behavioral Health University of Colorado Aurora, CO United States; 3 Department of Medicine, Division of Endocrinology, Metabolism, and Diabetes University of Colorado Aurora, CO United States; 4 Peer Mentored Care Collaborative School of Medicine University of Colorado Aurora, CO United States

**Keywords:** diabetes care, diabetes distress, primary care, healthcare chatbot, artificial intelligence, eConsult, care pathways, clinical workflows

## Abstract

**Background:**

Diabetes distress (DD) is a common emotional response to living with diabetes. If not addressed, DD can have negative impacts on diabetes management, including the progression to mental health conditions such as depression and anxiety. Routine screening and treatment for DD is recommended, with primary care being an ideal setting given that the majority of people with diabetes receive their diabetes care from primary care providers. However, consistent screening of DD does not routinely occur in primary care settings. Research is needed to understand how to effectively and feasibly integrate DD screening and treatment into routine diabetes care.

**Objective:**

This study aims to (1) design and implement individualized technology-supported DD workflows, (2) evaluate the primary outcome of determining the acceptability and feasibility of integrating technology-based workflows to provide treatment for DD, and (3) evaluate the secondary outcomes of changes in DD, depression, and anxiety (baseline, 3 months, and 6 months) in patients receiving screening and personalized treatment.

**Methods:**

In total, 30 English and Spanish-speaking primary care patients with either type 1 or type 2 diabetes will receive screening for DD during clinical visits and subsequent support from an artificial intelligence (AI)–based health care chatbot with interactive tailored messaging. In addition, the use of electronic consultation with a specialist or referral to a behavioral health provider could occur depending on the severity and source of DD. The use of electronic consultations allows providers convenient and timely asynchronous access to a range of specialty care providers. Health outcomes will be measured through changes in validated screening measures for DD, depression, and anxiety. Digital outcomes will be measured through surveys assessing user experience with technology and system usability, and by system performance data. Qualitative data on acceptability and satisfaction with the clinical workflows and technological interventions will be collected through interviews with patients and clinical providers. Descriptive statistics will summarize quantitative outcome measures and responses to closed-ended survey items, and rapid thematic and content analysis will be conducted on open-ended survey and interview data.

**Results:**

Workflows for screening and treating DD have been approved and clinical staff have received training on the process. Electronic surveys for screening measure collection have been created. Data from visit screeners will be entered into the electronic medical record during the medical appointment. Recruitment will begin late June-July 2024.

**Conclusions:**

This study is expected to demonstrate the feasibility and acceptability of integrating individualized workflows for DD into primary care. Improving clinical and digital interventions for addressing DD in primary care can provide alternative care options for busy clinical providers. This study is intended to deliver whole-person diabetes care to people with diabetes within a primary care setting.

**International Registered Report Identifier (IRRID):**

PRR1-10.2196/62916

## Introduction

### Backgrounds

In the United States, diabetes is the eighth leading cause of death [1], with an estimated 38 million people living with diabetes [1]. This chronic condition requires consistent care for effective management to help avoid poor health outcomes [2]. In fact, it is estimated that people with diabetes spend over 8000 hours per year managing their diabetes outside of medical settings [3]. Diabetes distress (DD) is the disruptive and demanding emotional response to these daily demands of living with diabetes [4]. This emotional burden associated with diabetes is pervasive, with one in 4 people experiencing severe DD [4]. DD is associated with negative impacts on engagement in self-care and self-management behaviors, medication adherence, and exacerbation of mental health conditions [5].

Accordingly, the American Diabetes Association recommends that diabetes care be delivered by an interdisciplinary team with a person-centered approach [6] and that it includes regular screening for and monitoring of DD in routine diabetes care for people with diabetes with treatment for DD to be provided by practitioners with specific training to address DD [5]. In addition, a recent white paper by the National Committee for Quality Assurance suggested having a variety of pathways to tailor DD treatment for individuals who screen positive for DD by involving relevant health care professionals and care modalities [7]. Yet, in everyday clinical settings, DD is infrequently identified and only a small number of people with diabetes are asked about how diabetes affects their life by their health care professionals [8]. As primary care is where most people receive their diabetes care, it is a crucial setting within which to assess and address DD [9,10]. However, there remains a lack of consistent screening for DD within primary care, which likely contributes to the emotional burden of diabetes going undetected and untreated [4,11]. Thus, even though DD is highly prevalent and there exist well-validated measures to assess for DD, there is a significant knowledge gap in best practices for implementing DD screening and treatment interventions systematically in primary care. Intervention studies specifically aimed to treat DD have largely been conducted in specialty care settings and not in primary care [5]. Further, among health care providers there remain a lack of awareness of DD and a shortage of trained health care providers who feel adequately equipped to assess for DD and intervene when a patient is experiencing DD [12]. Consequently, there is a clear need to develop scalable and feasible workflows and interventions that can be easily incorporated into primary care settings. Implementing assessment and treatment for DD is a crucial part of incorporating the psychological and emotional aspects of living with diabetes into routine diabetes care. It is part of intentionally incorporating the often-forgotten about psychological and emotional aspects of diabetes care that comprise whole-person care.

Clinical decision support systems (CDSS) have proven effective in prompting providers to deliver recommended care [13]. In general, CDSS improves health care delivery through the use of technology to enhance clinical decision-making, sometimes even using data and observations that are normally unobtainable by providers alone [14]. Clinical decision support technology leverages electronic health records (EHRs), medical knowledge databases, and algorithms to provide patient-specific recommendations, thus enabling providers to make more informed decisions [15]. Benefits can include a reduction in medical errors, enhanced patient safety, improved decision-making, and scalability [15]. The recent integration of artificial intelligence (AI) into health care technology has led to the classification of CDSS as either knowledge-based systems using traditional technology frameworks, or nonknowledge-based systems to indicate the use of AI to transform data into information for the user [16]. Recent reviews of studies that focus on the implementation of nonknowledge-based CDSS in diabetes care have demonstrated significant improvements in patients’ blood glucose, blood pressure, and lipid profiles in 71%, 67%, and 38% of the studies, respectively [16]. Technology-based referrals to specialty care electronic consultation (eConsults) have been shown to successfully augment and support care provided in the primary care setting without requiring patients to leave their medical home. Curated health chatbots are an interactive, patient-focused approach to providing patients with important information in an empathic manner at the time of need and point of inquiry, without requiring appointments or waiting for return calls from highly burdened health care professionals. While CDSS can promote diabetes care by facilitating patient self-management, it is hoped that further emerging technology will allow for more efficient and effective management for many people living with diabetes [16].

Research examining the treatment of DD indicates that when DD is specifically targeted that it can be improved, which is important given that DD is known to worsen over time if it is not addressed [17]. eHealth interventions providing support for DD have shown promising results. For instance, a systematic review and meta-analysis concluded that eHealth interventions were effective at significantly reducing DD and that these reductions occurred across a range of different eHealth interventions (eg, telehealth, web-based, and mobile health) [18]. While the eHealth interventions were found to reduce DD, the authors of the meta-analysis indicate the results be interpreted with caution because of the low number of studies included in the review [18]. Additional research delivering DD interventions through flexible and affordable eHealth avenues requires further exploration. This study is intended to build upon previous research suggesting that digital interventions could provide an avenue for treating DD.

### Objective and Aims

The primary objective of this study is to assess the feasibility and accessibility of using health IT integrated into primary care workflows to improve screening and treatment for DD. This project will examine the technical and operational feasibility, patient and provider experience, and behavioral health outcomes of a new technology-supported workflow to conduct screening for DD and provide follow-up treatment by a multidisciplinary team in a primary care setting.

The aims of this pilot study are to (1) design and implement individualized technology-supported DD workflows, (2) evaluate the acceptability and integration of technology-based workflows to provide treatment for DD, and (3) evaluate the change in DD (baseline, 3 months, and 6 months) in patients receiving screening and personalized treatment for it. Symptoms of anxiety and depression will also be evaluated. The primary outcome of this study is to determine the feasibility and acceptability of integrating individualized workflows for DD into primary care.

## Methods

### Intervention Implementation Process

#### Preimplementation

The principal investigator (PI) conducted a 1-hour practice-level training session for clinical team members including primary care practitioners, medical assistants, administrative staff, and behavioral health providers. The training topics included an orientation to DD, person-first language when working with people with diabetes, a review of the DD screeners, and a brief introduction on how to support patients experiencing DD. This training was informed by expertise from clinical experts on DD. The training also reviewed the clinical workflow for this study and provided the opportunity for staff and providers to ask questions about the protocol.

#### Implementation

**Figure 1:**
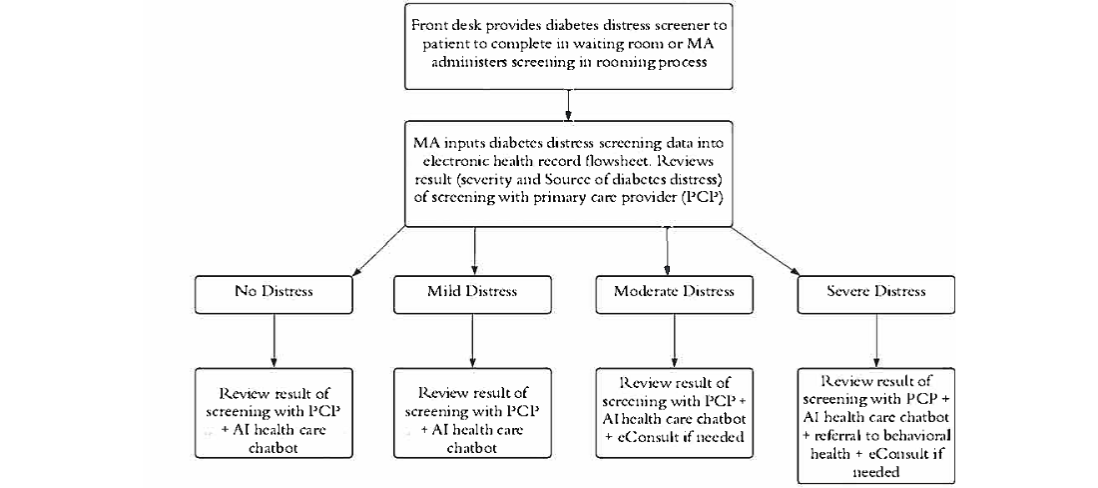
Primary care diabetes distress screening and treatment workflow. AI: artificial intelligence; eConsult: electronic consultation; MA: medical assistant; PCP: primary care physician.

Patients will complete informed consent to participate in the research study. After being consented, they will attend a scheduled diabetes-specific visit with their primary care physician (PCP). The workflow for the diabetes-specific visit is presented in Figure 1. At clinic check-in, front desk staff or a medical assistant (MA) will provide the patient with the appropriate assessment to complete based on their diagnosis, either the Type 1 Diabetes Distress Assessment System (T1-DDAS) [19] or the Type 2 Diabetes Distress Assessment System (T2-DDAS) [20]. Clinic staff will also provide the patient with the Patient Health Questionnaire-9 (PHQ-9) [21] to assess for symptoms of depression and the Generalized Anxiety Disorder-7 Scale (GAD-7) [22] to assess for symptoms of anxiety. The PHQ-9 and GAD-7 questionnaires are already part of the rooming process and thus not an addition to the established workflow. During the visit rooming process, the MA will enter the results of the screeners (eg, PHQ-9, GAD-7, T1-DDAS, or T2-DDAS) into the flowsheets in the EHR. The EHR used in this study is Epic. Refer to Table 1 for measure descriptions.

**Table 1:** Diabetes distress pilot study measures.

Measure	Description	Collection time points
T1-DDAS^a^ [19]	30 items; 5-point Likert scale. Assesses the emotional impact of living with type 1 diabetes. Determines the severity and Source of DD^b^. The sources of DD indicate which aspects of living with type 1 diabetes are creating challenges. The T1-DDAS was validated with adults with type 1 diabetes and has adequate internal consistency on the Core Scale (α=0.95) and Source Scales (α range=0.53-0.88).	Baseline (at initial clinic visit) 3 months post screening 6 months post screening
T2-DDAS^c^ [20]	29 items; 5-point Likert scale. Assesses the emotional impact of living with type 2 diabetes. Determines the severity and source of DD. The Sources of DD indicate which aspects of living with type 2 diabetes are creating challenges. The T2-DDAS was validated on people with type 2 diabetes including both insulin and noninsulin users. Adequate reliability for the Core scale was demonstrated by alpha statistics for noninsulin users (α=0.94) and insulin users (α=0.95). The Core Scale has statistically significant construct validity with the 7 Source Scale criterion variables (all, P<.001).	Baseline (at initial clinic visit) 3 months post screening 6 months post screening
PHQ-9^d^ [21]	9 items; 4-point Likert scale. Brief screener assessing symptoms of depression. Amongst a primary care population, the internal reliability was excellent (Cronbach α=0.89) [21]. The PHQ-8^e^ omits question 9 on the PHQ-9, which asks respondents about thoughts of self-harm or death.	Baseline (at initial clinic visit) 3 months post screening 6 months post screening
GAD-7^f^ [22]	7 items; 4-point Likert scale. Brief screener assessing symptoms of anxiety. The reliability of the weighted scoring of the items on the GAD-7 has been estimated at 0.91 with internal consistency estimated at 0.92.	Baseline (at initial clinic visit) 3 months post screening 6 months post screening
UMUX-lite^g^ [23]	2 items; 7-point Likert scale. Brief measure assessing participant’s experience of technology (ie, AI^h^ chatbot). Internal reliability estimates for the UMUX-lite range from 0.81-0.87).	3 months post screening
System Usability Scale [24]	10 items; 5-point Likert scale. Brief measure assessing the usability of the technology (ie, AI chatbot). Research demonstrates an average reliability using coefficient alpha to be 0.91. Concurrent validity ratings range from 0.22 to 0.96.	3 months post screening
Technology Use Assessment [25]	A 12-item survey assessing user’s comfort with and routine use of technology in daily life.	Baseline (at initial clinic visit) 3 months post screening
Practice demographics	Clinic data (ie, number of patients with diabetes, clinical staff, and roles).	Baseline
AI Chatbot System Performance Metrics	Engagement and operational metrics recording during the period of system use.	3 months post screening
eConsult^i^ System Performance Metrics	Operational metrics recorded during system use from study-specific standardized templates, including the number of patients receiving eConsults, which specialists were eConsulted, what were the consult questions, the time to respond by the specialist, and did the patient follow up with the specialist if referred.	3 months post screening 6 months post screening
Patient demographics	Includes age, race, ethnicity, gender, sexual orientation, health care insurance type, and comorbid medical diagnoses if applicable.	Baseline
Qualitative data-clinical staff	Semistructured interview and survey questions. Questions assessing experience implementing new workflows and screening patients for DD from clinical staff. A trained qualitative researcher will conduct interviews over the phone or video call according to the semistructured interview guide.	3 months post screening
Qualitative data-patients	Semistructured interview and survey questions. Questions assessing participants’ experience of being screened for DD, using an AI chatbot, and being referred to targeted providers using eConsults. A trained qualitative researcher will conduct interviews over the phone or video call according to the semistructured interview guide.	3 months post screening
Diabetes Distress Provider Time Survey	Survey form tracking the types of tasks and amount of time spent on tasks related to workflow.	Primary care physician completes following each participant encounter.

^a^T1-DDAS: Type 1 Diabetes Distress Assessment System.

^b^DD: diabetes distress.

^c^T2-DDAS: Type 2 Diabetes Distress Assessment System.

^d^PHQ-9: Patient Health Questionnaire-9.

^e^PHQ-8: Patient Health Questionnaire-8.

^f^GAD-7: Generalized Anxiety Disorder-7 Scale.

^g^UMUX-lite: Usability Metric for User Experience-lite.

^h^AI: artificial intelligence.

^i^eConsult: electronic consultation.

If there is a negative screen on the T1-DDAS or T2-DDAS, the MA will communicate this to the PCP. The PCP will then provide validation and encouragement based on the skills they learned in the team training. The patient and provider will then collaboratively determine which aspects of DD the patient would like to start to receive information and guidance on from the AI chatbot. Even though the patient is not currently experiencing DD, it is theorized that receiving coping skills and other information from the chatbot will help mitigate future distress. The PCP will send these identified areas through the EHR to the research team so they can send push notification from the AI chatbot to the patient’s cell phone in these specific areas.

If there is a positive screen on the T1-DDAS or T2-DDAS, the MA will communicate the severity and source of DD to the PCP. On the T1-DDAS there are 10 sources of distress including financial worries, interpersonal challenges, management difficulties, shame, hypoglycemia concerns, health care quality, lack of diabetes resources, technology challenges, burden to others, and worries about complications [19]. The T2-DDAS indicates specific areas or sources of distress that people with type 2 diabetes may experience [20]. On the T2-DDAS there are 7 sources of distress including hypoglycemia, long-term health, health care provider, interpersonal issues, shame or stigma, health care access, and management demands [20]. The research team has developed specific content areas for sources of distress for both the T1-DDAS and T2-DDAS meant to provide targeted and individualized material to support the needs of patients delivered through the AI chatbot. The study design for levels of DD is the same for type 1 and type 2 diabetes. There are three levels of distress on the T1-DDAS and T2-DDAS: (1) little or no distress, (2) moderate, and (3) high. The levels and source of distress will guide clinical decision-making and intervention pathways that are selected. For each level of distress, the PCP will provide validation and support to patients based on skills they learned during the team training. For little to no distress on the T1-DDAS or T2-DDAS, the PCP will discuss the top 3 sources of distress with the patient and the patient will then obtain a push notification on their cell phone from the AI chatbot on these specific areas. For moderate levels of DD on the T1-DDAS or T2-DDAS, the provider will discuss the main sources of distress with the patient and determine if an eConsult or referral might be indicated based on the needs of the patient in addition to the AI chatbot. An eConsult to the following specialists can include endocrinology, clinical pharmacy, social work or care management, diabetes education, and behavioral health. All these specialty providers are available in the primary care practice where this study will occur. For high levels of DD on the T1-DDAS or T2-DDAS, the PCP will collaboratively determine which areas of distress would be more relevant for the patient to receive from the AI chatbot and determine if an eConsult to any of the above-stated specialties would be indicated and then subsequently discuss and provide a referral to behavioral health if needed. Integrating eConsults into the treatment process allows the PCP to ask a specific clinically oriented question about their patient and obtain an asynchronous response from the specialist. Thus, allowing for shared care decisions between the PCP and specialist without having to refer the patient to an in-person appointment and leading to a possible delay in care [26].

#### AI Chatbot Intervention

All patient participants, regardless of whether they have DD or the severity of the DD, will be enrolled to receive text messages from the AI chatbot. The AI chatbot will provide education on DD, normalization of DD, suggestions for solution-focused coping strategies, and information provision on patient resources and support. For patients not reporting symptoms of DD, chatbot messaging will be seen as a preventative measure to provide information and build awareness of DD should this arise in the future. For patients reporting DD, the AI chatbot will be seen as a resource to provide suggestions for coping strategies connecting to local support resources and psychoeducational material. Following a collaborative discussion with their PCP on the sources of DD that they would like to receive support on, they will obtain a push notification with the top 3 identified areas of distress. Following this initial conversation, the AI chatbot will deliver a 12-week curriculum on topics specifically related to DD and the sources of DD (for either the T1-DDAS or T2-DDAS). Participants will receive 3 scheduled text messages on the curriculum content per week throughout the 12 weeks to their cell phone. In between scheduled messages, participants can initiate interaction with the AI chatbot through the text message chain if they choose. Development of the AI chatbot content was overseen and reviewed by DD expert consultants. Participants will not receive messages on topics unrelated to DD or content that was not developed and approved by the study team. In other words, participants will not receive content that is generated by the system or off-topic.

The technology will facilitate error-free delivery of messages via text to user cell phones using an AI chatbot that deploys natural language processing for highly precise communications. We will maximize chatbot precision so that users are more often sent a response from our system that matches the intent of their query. Specifically, we have developed and categorized anticipated “intents”—that is, the specific topics we believe people want to learn or ask about DD and self-management, along with 25-50 variations on ways to ask each question. Question variations allow the system to have enough initial data to learn how to interpret user questions, tolerate misspellings, and recognize the underlying intent of each question. When the system cannot match a response to the question intent, it reverts to a fixed choice (called a “pick list”) of responses, for example, “I think you are asking about one of these topics: (1) Cost of diabetes medicine, (2) Cost of treatment, (3) Where to find medications near me. Please type the number corresponding to the topic you wish to explore or try your question again.” We rely on data augmentation techniques to create and continuously update a robust library of questions and question variations that the system draws on to generate precise and consistent responses to user queries. We do this through “lemmatization” and “stemming,” both processes that group the different inflected forms and stems of a word so they can be analyzed as a single item (eg, runs, run, and running are all forms of the word “run” and thus “run” is the lemma, or root, for all these words). After doing this preprocess work on our prototype dataset, we use Multinomial Naïve Bayes, Linear SVC, and multiclass regression algorithms to anticipate prediction accuracy in correctly matching a response to a question.

#### eConsult Intervention

The EHR-embedded eConsult system provides a streamlined and timely consultative process that has been shown to improve the quadruple aim-related outcomes and to enhance communication and coordination of care between primary care providers and specialists regarding specific patient areas of concern [27]. The PCP sends a focused question with relevant subjective and objective patient information to a specialist through the eConsult system. The specialist then reviews pertinent information from the EHR and responds to the PCP with guidance and care support with recommendations regarding diagnosis, treatment, and follow-up plans.

Following the patient-specific interaction with the PCP in the diabetes-specific visit, an eConsult with different specialties (indicated above) will be placed through the EHR. The eConsult system is already an established part of routine clinical care since it is embedded in the EHR. eConsults will be used to assist with clinical decision-making and obtaining specialized knowledge and support from various health care professionals based on the specific source of DD. If the patient requires additional support, the eConsult system will allow for a conversion to an in-person or telemedicine visit with the needed specialty.

#### Poststudy Intervention

Poststudy time point 1 (3 months following initial screening): the research assistant (RA) will send patients a link to complete the screening measures through the patient portal. The screening measures completed at 3 months post screening will assess for DD, symptoms of depression and anxiety, and technology use (refer to Table 1). Outside the clinical setting, the PHQ-8 will be administered instead of the PHQ-9 [28]. The technology measures include the Usability Metric for User Experience-lite (UMUX-lite) [23], System Usability Scale (SUS) [24], technology use assessment, AI chatbot system performance metrics, and eConsult system performance metrics. Qualitative data will be collected at 3 months post screening. Table 1 includes the measures and time points for data collection. The RA will call each patient to conduct a semistructured interview and ask survey questions if patients have not completed surveys electronically within 1 week. The qualitative interview will include questions about participating in screening for DD, experience with the AI health care chatbot and being referred to specialty providers through eConsults. Further, the RA will obtain qualitative data through semistructured interviews and survey questions with 6 clinical staff and both primary care providers who participated in the study. The questions for health care staff or providers will assess their time spent and experience implementing workflows that screen for DD and offer treatment options through provider support, AI health care chatbot, and eConsults.

Poststudy time point 2 (6 months following initial clinic visit): the RA will send patients a link to complete the screening measures through the patient portal at the 6-month time point. The screening measures completed at the 6-month time point are included in Table 1. If patients do not complete the surveys electronically within 1 week, the RA will call to follow up and administer surveys via phone.

### Study Design

We propose a pilot clinical trial to be conducted at a suburban multidisciplinary family medicine practice in an academic medical setting. The study is designed to provide feasibility and acceptability data for the development of DD-based screening and treatment using digital and clinical interventions.

### Participants

Up to 30 adult English- or Spanish-speaking patient participants with a diagnosis of type 1 diabetes or type 2 diabetes who receive their diabetes care from 2 PCPs at the primary care practice will be enrolled in the study. For Spanish-speaking participants, Qualified medical interpreters are used for translation services that are provided at no cost at the clinic. Qualified medical interpreters are part of routine medical services. A sample size of 30 is the minimum generally recommended if the underlying population is expected to have a normal distribution [29]. It is also double the minimum recommended number of persons to be included in human factors and usability testing for medical devices, which allows for loss to follow-up or nonparticipation by some individuals in data collection activities [30].

Clinic staff engaged in the new workflows for DD will also be invited to participate in surveys and interviews about their experience with the technology-supported intervention following the completion of the study. Inclusion and exclusion criteria are mentioned in Textbox 1.

**Inclusion and exclusion criteria.**

**Inclusion criteria**

- Age at time of consent 18-89 years;
- Diagnosed with type 1 or type 2 diabetes;
- Patient at the primary care clinic;
- Able to understand English or Spanish;
- Willing and able to sign the informed consent form;
- Willing to be contacted by the study team through the patient portal, phone, or text to complete study measures;
- Ability to reliably send and receive text messages.

**Exclusion criteria**

- Participation in another study that might interfere with participation in this study;
- Unable to follow the study procedures for the duration of the study or is deemed unacceptable to participate in the study per principal investigator judgment;
- Participant or participant’s immediate family member is an employee of the health care chatbot company providing services for the study;
- Planning to move in the next 6 months;
- Planning to change primary care practices in the next 6 months.

### Data Collection and Outcome Measures

Both qualitative and quantitative methods will be used to assess study outcomes at the patient level, practice level, and technology system level. Patient-level health outcomes include DD, depression, and anxiety measured at baseline, 3 months, and 6 months using the T1-DDAS or T2-DDAS, PHQ-8, and GAD-7. The T1-DDAS was selected to be used in this study because it is the most updated and comprehensive assessment tool for determining DD in people with type 1 diabetes. The T2-DDAS was used as a measure because it is the most comprehensive and contemporary survey tool to determine DD among people with type 2 diabetes. The PHQ-9 and GAD-7 are commonly used brief screeners in primary care [21,22]. User experience with technology will be assessed through the administration of the UMUX-lite at 3 months, the SUS at 3 months, and a technology use assessment at baseline and 3 months. The SUS is widely adopted as a reliable measure of usability across technology types and in a variety of populations. The UMUX-lite represents a user experience-focused usability measure that has proven to correlate well with the SUS in overall usability and is appropriate for use with health technologies. The technology use assessment was previously developed by a member of the research team for use in discerning technology adoption and use patterns with primary care patients [25]. In addition, qualitative data from interviews and responses to open-ended survey items will be collected from practice staff, PCPs, and patients to determine the acceptability and feasibility of implementing screening and technology-supported treatment for DD. Interviews will be conducted by a trained qualitative analyst according to a semistructured interview guide, recorded and transcribed using artificially intelligent transcription software (Otter.ai), with human adjudication of transcripts to avoid error (refer to Multimedia Appendices 1 and 2 for interview guides). Positive experiences with the digital interventions will be determined by participant responses to the qualitative interview questions. Further, PCPs will complete the DD Provider Time Survey to track activities and time spent on workflow tasks. Acceptability with the eConsults will be evaluated by standard data collection protocols in the EHR. eConsult data will capture the patient-specific inquiries and requests from PCPs and responses given by specialists. AI chatbot performance data will be collected from the chatbot system and used to evaluate engagement with the AI chatbot according to the People at the Center for Mobile Application Design framework [31]. Table 1 lists the measures to be collected for this study in detail.

A number of the validated measures have existing published versions in both English and Spanish including the T1-DDAS, T2-DDAS, PHQ-8, and GAD-7. For outcomes measures including the UMUX-lite, SUS, and the technology use assessment that did not have an available version in Spanish, we had a system-verified language translator assist in translating these materials from English into Spanish.

### Data Analysis

#### Quantitative Data Analysis

The study team will use standard statistical packages (eg, R [R Foundation for Statistical Computing]) to conduct data analysis. Descriptive statistics (means, SD, frequency distributions, and proportions) will be used to summarize baseline patient characteristics, clinical and behavioral outcomes, and other quantitative outcome measures and responses to closed-ended survey items. As a feasibility study, it is not powered for inferential analyses or power analyses, but the results are expected to inform future work. Based on results from similar studies, a 60% (18/30) threshold will be used to determine technical and operational feasibility and user acceptability, that is, 60% (18/30) of participants remain engaged with the technology solutions throughout the duration of the study, and 60% (18/30) of participants report positive experiences and satisfaction levels with the program overall [32]. The study has been planned to minimize the amount of missing data overall and thus anticipates the use of maximum likelihood methods to estimate missing values for statistical analysis. We will use this data to calculate an effect size to power a larger study.

#### Qualitative Data Analysis

This study’s acceptance is determined by patient and health care provider user experience through interviews, clinical team member feedback, and responses to user experience surveys. An overall positive rating on tailored survey items or positive themes identified from qualitative data represents acceptability for this study. The program will be deemed acceptable among participants overall if 60% (18/30) or more of participants report positive ratings and themes. Qualitative analysis of open-ended survey data and interview data will be conducted using rapid thematic and content analysis to assess user experience by identifying and exploring common topics and themes that emerge from participants’ responses. The rapid analysis will be informed by the Consolidated Framework for Implementation Research and will use a dual-read approach and matrix classification method with 2 reviewers, who will discuss classification decisions to achieve consensus [33]. Enough participants are included in the planned sample size to ensure qualitative analyses will reach thematic saturation overall.

### Ethical Considerations

#### Approval and Study Consent

This study has been approved by the Colorado Multiple Institutional Review Board (protocol #24-0186). The PI and primary care providers will assemble a patient list from the Diabetes Registry in the EHR and send this to the RA. The RA will outreach to potential participants via the patient portal in the electronic medical record, by email, and by phone using scripted language to invite patients to participate. As part of enrollment, the patient will opt-in to receive text messages from the AI chatbot. If a patient declines to participate, this will be documented for evaluation purposes so that the same patient is not reapproached in association with a future scheduled clinic visit. If the patient expresses interest in participating, the research team will complete informed consent and study enrollment before a future clinic visit. As part of the consent process, patients will agree to a chart audit by the research assistant within 4 weeks of their initial visit to ensure all initial measures are completed.

#### Safety and Potential Risks

The procedures used in the proposed research study pose no greater than minimal risks to patients and practices involved in the study. The AI chatbot is a nongenerative system meaning that it does not independently develop content. The primary clinical team has generated the content and reviewed it with expert consultants on DD. Further, no Protected Health Information is requested from patient participants or sent from the AI chatbot. The AI chatbot operates on HIPAA (Health Insurance Portability and Accountability Act)-compliant infrastructure under a business associate agreement between the health care system where this study is taking place and the AI chatbot company. During the course of this study, patients could experience worsening of their symptoms of DD as a result of interacting with the AI chatbot and discussing the emotional and psychological aspects of diabetes with their medical provider. However, we do not expect that these are likely risks for patients participating in the study in part because messages provided by the chatbot have been prewritten and approved by clinicians and the chatbot is not using in-the-moment generative AI technologies to respond, such that it cannot create separate content or go off-script. If these concerns arise through a review of messages and chatbot interactions or as reported by patients, the PI will review and refer as appropriate to other health care providers for follow-up. The clinic where this study will take place has fully staffed behavioral health providers integrated into the practice who will be available to discuss any concerns that providers and patients may experience.

#### Compensation

Patient participants will receive a small financial incentive at 2 separate time points. They will receive two US $25 gift cards (totaling US $50). The first US $25 gift card will be given after enrollment in the study and completing the first set of surveys. The second US $25 gift card will be given after completing the 3-month postscreening questionnaires. Providers will receive no direct financial incentive. The primary care clinic where this study will take place will receive a financial incentive for participating in and attending the team training.

## Results

### Implementation Status

The PI met with clinic leadership to describe the project and obtained buy-in from the team. Workflows were developed that outline study design and patient flow (refer to Figure 1). The PI conducted a team training with the clinic where this study will take place as well as met with the PCPs that will be participating in this study to provide education and training on DD, an overview of validated measures for DD (eg, T1-DDAS and T2-DDAS) and conversational tools that can be used to support people with diabetes and DD.

T1-DDAS and T2-DDAS scoring were incorporated into the EHR through an EHR build. The PI collaborated with the EHR administrative team on requesting the 2 DD measures be incorporated into the EHR. This was done during the annual optimization and training period, namely the Epic Sprint program [34]. This program is intended to improve the users’ experience of the EHR and assist with updates and requests. The DD measures were built into the EHR as flowsheets so that data could be entered and scored in the EHR.

Chatbot content specific to DD was developed with assistance from 2 leading diabetes psychologists with expertise in DD. The AI chatbot was field tested by the primary research team and the project was reviewed with patients with diabetes through a patient advisory committee including 8 members and feedback was incorporated in refinements of the chatbot content. Key feedback that was incorporated included the frequency of chatbot messages and the time of day that chatbot messages would be sent. The primary research team field-tested the chatbot by sending and receiving text messages to their cellular phones and documented any errors, challenges, or missing content that they experienced. This information was reviewed and discussed with the chatbot vendor and incorporated into the chatbot analytics and content library. The field testing and feedback process was repeated twice.

### Recruitment Status

Patient recruitment is anticipated to begin during late June-July 2024. Recruitment will likely take place through January 2025.

### Data Collection Timeline

We anticipate that data collection will be completed by July 2025.

### Research Status

Institutional review board approval was obtained on March 15, 2024. We have signed contracts with RAs that will be performing the duties of providing outreach to eligible patients, obtaining informed consent from patient participants, and administering screening tools at the 3-month postscreening and 6-month postscreening time points. In addition, RAs will ensure that data collection from patient screeners is complete and documented appropriately. Following 3 months post screening, research assistants will conduct the semistructured interviews and administer the survey questions to both patients and clinical staff. At the 6-month study time point, the primary team will send the remaining screeners to patient-participants. The primary research team has been meeting weekly since November 2023 to develop the research plan and discuss project tasks.

### Technology Status

Licensing agreements and institutional risk assessment approval for the AI chatbot were obtained before patient recruitment. The AI chatbot was initially beta-tested by the primary research team and patients with diabetes to determine if messages could be delivered on a schedule and if the system could get replies back. The beta test revealed that additional content related to suicidality and “hating having diabetes” was needed, more training on the model to correctly match intents to the content library was needed, and that more of the intents had existing content in the chatbot library but that improving the link to these was still needed. The clinical research team developed content related to suicidality including providing crisis resources and directions to seek emergency services if needed. The introductory chatbot disclosure message was reviewed and indicated that the chatbot is not a replacement for medical treatment and to contact 911 for medical emergencies. Before the study launch, the primary research team retested the chatbot system to ensure that the updates had been completed.

eConsults are an active clinical care option for PCPs at our institution, fully integrated into the EHR and in use by over 28 specialties [35]. As a result, they are a readily usable aspect of this study. For our study, specialists available by eConsults will include behavioral health, social work, care management, diabetes education, pharmacy, and endocrinology.

T1-DDAS and T2-DDAS were created and incorporated into the EHR as flowsheets. The PHQ-9 and GAD-7 are already embedded in the EHR. The results of the DD screeners will be able to be pulled into visit documentation, facilitating care coordination with eConsulted providers. In addition, flowsheet data can be tracked over time and will be easily accessible for providers to review during and after patient visits.

The chatbot performance metrics will be monitored throughout the course of this study and evaluation of the chatbot functionality is part of the feasibility. The study team will monitor for communication failures and in the event of longer-term impact will contact participants directly.

### Funding Status

Funding for this study was secured from the Peer Mentored Care Collaborative in January 2024. The duration of the funding was 1 year.

## Discussion

### Expected Findings

This pilot study is expected to demonstrate the acceptability and feasibility of implementing screening and treatment for DD in a primary care clinic. Through this study, workflows will be developed and implemented to screen for DD at a diabetes-specific visit. Following screening for DD, patients will engage in a clinical conversation with their PCP about the results of the screening measure. A tiered approach will be used to determine the type of intervention that is suggested to the patient. All patients (regardless of DD screening result) will receive access to the AI chatbot. Patients might also receive an eConsult with another health care specialist (eg, social work, clinical pharmacy, care management, behavioral health, endocrinology, and diabetes educator) to support the specific Source of DD. If levels of DD are in the high range, patients may also receive a referral to a behavioral health provider.

We anticipate that the use of technological interventions such as the AI chatbot and eConsults will be experienced positively by participants. It is expected that participants will view the AI chatbot as beneficial to their diabetes care and will find it easy to use. The semistructured interviews will specifically request feedback on participant experience using the AI chatbot. The use of eConsults will allow PCPs to increase collaborative communication among the interdisciplinary team and provide participants with additional specialty services if needed. Having increased coordination among their primary care team will likely be seen as beneficial and helpful to participants. Existing research indicates that specifically targeting DD for intervention has positive benefits on the severity of distress [17]. Further, eHealth interventions for DD have been shown to be effective for improving diabetes-related distress [18].

This study is expected to increase provider and staff awareness and knowledge of screening for and intervening with DD through team and provider training. The training will provide opportunities for learning important psychological and emotional aspects of patients with diabetes that often go overlooked in clinical care. Clinical conversations focusing on the emotional side of living with diabetes are expected to be a new experience for patients in this study. While this might be a new type of clinical interaction, obtaining validation, normalization, reassurance, and empathy from their medical provider will likely be seen as a helpful and rewarding experience. Living with diabetes is a challenging undertaking that under the best of situations requires nuanced interventions from providers and constant attention and monitoring on behalf of patients and their caregivers. Having a supportive environment to discuss challenging lived experiences is intended to improve the emotional burden of diabetes. This finding is anticipated given that emerging evidence suggests that how health care members speak to people with diabetes can impact the development or exacerbation of DD [12].

Even though DD has become increasingly viewed as an important aspect of diabetes care, there remains limited data on treatment approaches. Current literature points to the importance of addressing DD but interventions specifically focused on DD are needed [36]. Most studies do not examine DD as a primary outcome but deliver interventions focused on managing diabetes more broadly [36,37]. This study will provide interventions that are meant to target DD specifically. The primary research team intends to continue pursuing funding opportunities to conduct larger studies in DD assessment and treatment in primary care settings. Future research would expand the assessment and treatment of DD to entire practices involving the whole care team to provide consistency in whole-person diabetes care delivery.

DD significantly impacts clinical, behavioral, and psychosocial outcomes in people living with diabetes. The majority of people with diabetes receive their diabetes care in primary care. However, assessment and treating DD do not routinely occur in primary care settings. The use of several interventions, including supportive dialogue with PCPs, an AI chatbot, and eConsults, will assist in delivering individualized treatment and support for DD without contributing to increased workload for primary care practices. Learning how to provide screening and treatment for DD in primary care settings is crucial to improving the care of people living with diabetes. Health care systems should cultivate environments that promote open discussions about emotional health. This may include training staff to establish a welcoming atmosphere, integrating whole-person considerations into standard assessments, and ensuring that patients feel at ease when sharing their experiences. Embedding the assessment and treatment for DD within primary care workflows is intended to invite providers and patients to consider the broader impact of living with diabetes.

### Limitations and Challenges

Given the current state of rapid evolution for AI technologies and associated policies governing AI use in practice, obtaining institutional approval to use AI chatbot technology as part of patient care requires an extensive review process. While health care technology continues to be seen as a scalable and feasible form of care delivery, administrative barriers can make implementation and usage challenging. Overcoming some of these barriers will likely contribute to increased uptake of technology.

Potential barriers to the implementation of new interventions into primary care practices can include lack of leadership buy-in, lack of communication regarding the rationale for the intervention to all practice staff and providers, and workflow barriers. We have thoughtfully approached each of these barriers through our preimplementation work highlighted above, including practice-level training. The PI also met with clinic and health system leadership before the start of the study to secure support for the project. In addition, the study was designed to seamlessly integrate into already existing practice workflows that were being used for depression and anxiety screening. The integration of DD measures into the EHR was intended to further reduce implementation barriers.

Potential barriers to implementing our protocol in other primary care settings include differing resources. Access to eConsults is not universal and alternative protocols would need to be developed depending on practice settings. Furthermore, not all clinics have integrated behavioral health teams easily accessible. It is likely that providers and staff in other primary care settings may also require training on the diagnosis of DD given its emerging status within the spectrum of care for patients with diabetes. The results of this study could help inform what resources (eg, personnel and training) would be needed to successfully integrate DD assessment and treatment into diverse primary care practices.

A potential limitation of eConsult use is the increased, noncompensated workload on primary care providers, who must act on the e-consultant’s recommendations for each patient. Primary care is a fast-paced setting that is often stretched for resources, staffing, and time. Further, primary care is a diverse setting with varied access and structures to care delivery. Consequently, it is unlikely that a single approach or method of care is appropriate in all primary care settings. With significant variability in primary care, interventions need to have adequate flexibility to have a chance to be incorporated successfully into this care environment. Feasibility studies offer an opportunity to determine if interventions can be flexibly disseminated into primary care in a scalable fashion. For the AI chatbot, we anticipate a limitation to be the imperfect matching of responses to participant questions. We are mitigating this limitation by setting expectations upfront with participants and using the dataset to iteratively improve chatbot responses over time with use. We chose this curated content route versus an open generative AI approach to minimize potential risks from chatbot hallucination or inaccurate responses.

Given that this is a feasibility study with a small sample size, and we are not powered for inferential analyses, the results of this study will not be generalizable. However, the results of this study will be used as preliminary data for a larger trial.

### Conclusions

Creating and disseminating workflows for screening and treating DD in primary care is an important component for delivering whole-person diabetes care. The use of an AI chatbot to deliver individualized treatment and support for DD and eConsults providing additional specialty support are expected to help increase support and treatment for DD without contributing to an increased workload for primary care practices. This study is intended to help us begin to understand how to implement DD screening and treatment in primary care settings in a scalable and real-world manner.

## Appendix

Multimedia Appendix 1 In-depth interview with patients.

Multimedia Appendix 2 In-depth interview with providers/staff.

## Abbreviations

AI: artificial intelligence
CDSS: clinical decision support systems
DD: diabetes distress
eConsult: electronic consultation
EHR: electronic health record
GAD-7: Generalized Anxiety Disorder-7 Scale
HIPAA: Health Insurance Portability and Accountability Act
MA: medical assistant
PCP: primary care physician
PHQ-9: Patient Health Questionnaire
PI: principal investigator
RA: research assistant
SUS: System Usability Scale
T1-DDAS: Type 1 Diabetes Distress Assessment System
T2-DDAS: Type 2 Diabetes Distress Assessment System
UMUX-lite: Usability Metric for User Experience-lite

## Data Availability

Data sharing is not applicable to this article as no datasets were generated or analyzed during this study.
