# Correction: Assessment and Intervention for Diabetes Distress in Primary Care Using Clinical and Technological Interventions: Protocol for a Single-Arm Pilot Trial

**DOI:** 10.2196/75984

**Published:** 2025-04-23

**Authors:** Marisa Kostiuk, Susan L Moore, E Seth Kramer, Joshua Felton Gilens, Ashwin Sarwal, David Saxon, John F Thomas, Tamara K Oser

**Affiliations:** 1 Department of Family Medicine School of Medicine University of Colorado Aurora, CO United States; 2 Colorado School of Public Health Department of Community & Behavioral Health University of Colorado Aurora, CO United States; 3 Department of Medicine Division of Endocrinology, Metabolism, and Diabetes University of Colorado Aurora, CO United States; 4 Peer Mentored Care Collaborative School of Medicine University of Colorado Aurora, CO United States

In “Assessment and Intervention for Diabetes Distress in Primary Care Using Clinical and Technological Interventions: Protocol for a Single-Arm Pilot Trial” (JMIR Res Protoc 2025; 14:e62916) the authors made one addition.

An Acknowledgements section was added to the paper as follows:

We wish to acknowledge colleagues Sheana Bull and Adam Salyers at Clinic Chat, LLC, who developed the chatbot technology described in the manuscript.

The correction will appear in the online version of the paper on the JMIR Publications website, together with the publication of this correction notice. Because this was made after submission to PubMed, PubMed Central, and other full-text repositories, the corrected article has also been resubmitted to those repositories.

