# Supplementary material for: Assessment and Intervention for Diabetes Distress in Primary Care Using Clinical and Technological Interventions: Protocol for a Single-Arm Pilot Trial
Source: JMIR Res Protoc. 2025 Mar 31;14:e62916. doi: 10.2196/62916 (PMC11997534; doi:10.2196/62916)
Supplement: Multimedia Appendix 1 [file resprot_v14i1e62916_app1.docx]

**In-Depth Interview with Patients**

Introduction

Thank you for joining us today.

My name is [NAME], and I’m part of the research team at the [STUDY SITE]. We want to thank you for being part of this research study on how to use technology to help support patients who might experience distress about diabetes, and we want to learn about your experience with the screening survey and chatbot system that you used as part of this research study.

Today’s interview will last about 30 minutes. We very much appreciate you taking the time and effort to talk with us and provide feedback on the survey and chatbot.

I want you to know that participating in this conversation is voluntary and you are free to stop the interview at any time. We take your privacy very seriously. With your permission, we will record the interview and we will make sure that your responses will not be associated with your name in any report. Do you have any questions?

[Confirm approval; begin recording.]

Questions

**In-Depth Interview with Patients**

1. As part of the study, you have been asked questions about the emotional and stressful aspects of living with diabetes. Could you please tell us about what your experience was like answering those questions?

Probes:

- 1. Did the questions *make sense* to you? Why or why not?
  2. Did the questions *seem like they applied* to you? Why or why not?
  3. Was it easy or hard for you to answer questions like this? Can you tell me a little bit about why?

1. Do you feel like answering these questions about diabetes distress made a difference in your care? Why or why not?
2. As part of the study, you have been asked to send and receive text messages from an artificially intelligent health care chatbot. Could you please tell us about your experience with the chatbot in the past 3 months?
   1. What kind of information did you receive from the chatbot?
   2. Was the chatbot easy to use? Why or why not?
   3. What did you like about sending or receiving messages from the chatbot?
   4. What did you dislike about sending or receiving messages from the chatbot?
   5. Did you have any difficulties with the chatbot? What things need to be improved?
   6. Would you recommend a friend with diabetes use a chatbot like this? Why or why not?

[Prompt for examples and details throughout.]

1. Did you start using a Continuous Glucose Monitor (CGM) at any time during your participation in this study? If yes, which CGM did you start using and when did you start using it?
2. Did you find the diabetes distress screening and chatbot support program beneficial for you in comparison to your normal care without the program? Why or why not?

[Prompt for examples: Can you tell me more about that? Can you give me an example of something that was beneficial/helpful/easier/assuring/necessary/burdensome/difficult?]

1. What concerns did you have in being part of the diabetes distress screening and chatbot support program?

[Prompt for examples: Can you tell me more about that? Can you give me an example of how?]

1. Is there anything that would make the diabetes distress screening and chatbot support program overall easier for you?

[Prompt to ask if they need any additional resources or support]

1. Do you have other ideas for how we could make care using this program better? Do you have any other thoughts you’d like to share?

This is the end of the interview. Thank you very much for your time and participation!
