# Supplementary material for: Assessment and Intervention for Diabetes Distress in Primary Care Using Clinical and Technological Interventions: Protocol for a Single-Arm Pilot Trial
Source: JMIR Res Protoc. 2025 Mar 31;14:e62916. doi: 10.2196/62916 (PMC11997534; doi:10.2196/62916)
Supplement: Multimedia Appendix 2 [file resprot_v14i1e62916_app2.docx]

**In-Depth Interview with Providers/Staff**

Introduction

Thank you for joining us today.

My name is [NAME], and I’m part of the research team at the [STUDY SITE]. We want to thank you for being part of this research study on how to use technology to help support patients who might experience distress about diabetes, and we want to learn about your experience with the clinic workflow and screening survey that were implemented as part of this research study.

Today’s interview will last about 30 minutes. We very much appreciate you taking the time and effort to talk with us and provide feedback on the study.

I want you to know that participating in this conversation is voluntary and you are free to stop the interview at any time. We take your privacy very seriously. With your permission, we will record the interview and we will make sure that your responses will not be associated with your name in any report. Do you have any questions?

[Confirm approval; begin recording.]

Questions

1. Can you tell me what your role is in the clinic?
2. As part of the study, can you explain the impact that screening for diabetes distress had on the clinical workflow?

Probes:

2.1 How feasible do you believe **assessing for** diabetes distress is?

2.2 How feasible do you believe **intervening with** diabetes distress is?

2.3 What would have made screening for diabetes distress easier for you in your role?

2.4 How did patients seem to respond to completing a screening measure for diabetes distress? (e.g., were there comments/questions about the length or types of questions on the screener?)

2.5 What aspects of assessing for diabetes distress seemed burdensome to the clinical workflow?

1. For those participants in the study that did not report experiencing any diabetes distress, what did your interaction look like? Tell me how you approached the conversation related to diabetes distress.
2. As part of the study, participants were asked to send and receive text messages from an artificially intelligent healthcare chatbot. Did you have discussions with participants about this aspect of the study? If yes: Please tell me about how those conversations went. (If no, continue to next question).

Probes:

4.1 What concerns did participants express about the chatbot?

4.2 What curiosity or points of interest did participants share about the chatbot?

4.3 Can you describe any difficulties that participants had with the chatbot, or any suggestions for improvement participants had?

1. As part of the diabetes distress study, participants might have been given a referral to a specialty provider such as social work, diabetes education, case management, clinical pharmacy, endocrinology, or behavioral health. Did participating in this study change your referral patterns to these specialty providers for patients with diabetes? [Depending on the clinical role this might be N/A].

Probes:

5.1 What concerns did participants express about seeing a specialty provider in reference to diabetes distress?

1. Tell me what you think the benefits to continuing to assess and provide intervention for diabetes distress would be after the study is over?
2. Tell me what you think the drawbacks to continuing to assess and provide intervention for diabetes distress might be after the study is over?
3. What are additional ideas for how we could make assessment and/or intervening for diabetes distress better? Are there any other thoughts you’d like to share?

This is the end of the interview. Thank you very much for your time and participation!
